# Tick parasites of rodents in Romania: host preferences, community structure and geographical distribution

**DOI:** 10.1186/1756-3305-5-266

**Published:** 2012-11-21

**Authors:** Andrei D Mihalca, Mirabela O Dumitrache, Attila D Sándor, Cristian Magdaş, Miruna Oltean, Adriana Györke, Ioana A Matei, Angela Ionică, Gianluca D’Amico, Vasile Cozma, Călin M Gherman

**Affiliations:** 1 University of Agricultural Sciences and Veterinary Medicine, Faculty of Veterinary Medicine, Department of Parasitology and Parasitic Diseases, Calea Mănăștur 3-5, Cluj-Napoca, 400372, Romania

**Keywords:** Hard-ticks, Ixodidae, Rodents, Micromammals, Romania

## Abstract

**Background:**

Ticks are among the most important vectors of zoonotic diseases in temperate regions of Europe, with widespread distribution and high densities, posing an important medical risk. Most ticks feed on a variety of progressively larger hosts, with a large number of small mammal species typically harbouring primarily the immature stages. However, there are certain Ixodidae that characteristically attack micromammals also during their adult stage. Rodents are widespread hosts of ticks, important vectors and competent reservoirs of tick-borne pathogens. Micromammal-tick associations have been poorly studied in Romania, and our manuscript shows the results of a large scale study on tick infestation epidemiology in rodents from Romania.

**Methods:**

Rodents were caught using snap-traps in a variety of habitats in Romania, between May 2010 and November 2011. Ticks were individually collected from these rodents and identified to species and development stage. Frequency, mean intensity, prevalence and its 95% confidence intervals were calculated using the EpiInfo 2000 software. A *p* value of <0.05 was considered statistically significant.

**Results:**

We examined 423 rodents (12 species) collected from six counties in Romania for the presence of ticks. Each collected tick was identified to species level and the following epidemiological parameters were calculated: prevalence, mean intensity and mean abundance. The total number of ticks collected from rodents was 483, with eight species identified: *Ixodes ricinus*, *I. redikorzevi*, *I. apronophorus*, *I. trianguliceps*, *I. laguri*, *Dermacentor marginatus*, *Rhipicephalus sanguineus* and *Haemaphysalis sulcata*. The overall prevalence of tick infestation was 29.55%, with a mean intensity of 3.86 and a mean abundance of 1.14. Only two polyspecific infestations were found: *I. ricinus* + *I. redikorzevi* and *I. ricinus* + *D. marginatus*.

**Conclusions:**

Our study showed a relatively high diversity of ticks parasitizing rodents in Romania. The most common tick in rodents was *I. ricinus*, followed by *I. redikorzevi*. Certain rodents seem to host a significantly higher number of tick species than others, the most important within this view being *Apodemus flavicollis* and *Microtus arvalis*. The same applies for the overall prevalence of tick parasitism, with some species more commonly infected (*M. arvalis*, *A. uralensis*, *A. flavicollis* and *M. glareolus*) than others. Two rodent species (*Mus musculus*, *Rattus norvegicus*) did not harbour ticks at all. Based on our results we may assert that rodents generally can act as good indicators for assessing the distribution of certain tick species.

## Background

Rodents (Order Rodentia) are usually small-sized mammals with a worldwide distribution, accounting for over 40% of all mammal species. Rodents are both widespread and abundant, as are their associated ticks. Thus, mainly from a human health perspective, the rodent-tick associations have a huge importance in most ecosystems [1]. Besides their role as tick hosts, rodents serve as reservoirs of tick-borne pathogens, hence increasing their importance in the eco-epidemiology of diseases like Lyme borreliosis, rickettsiosis, babesiosis, ehrlichiosis or tularaemia [1-3].

Most of the hard ticks feeding on rodents follow a three-host life cycle (i.e. each of the active stages - larva, nymph and adult - feeds on a different host individual). Usually, these ticks feed on a variety of progressively larger hosts, meaning that a large number of small mammal species typically harbour the immature stages [1]*.* On the other hand, there are certain Ixodidae that characteristically attack micromammals also during their adult stage. One of the most comprehensive reviews on micromammal-tick associations [1] lists 14 species of adult Ixodidae parasitic on rodents (*Anomalohimalaya cricetuli, A. lama, A. lotozskyi, Haemaphysalis verticalis, Ixodes angustus, I. apronophorus, I. crenulatus, I. laguri, I. nipponensis, I. occultus, I. pomerantzevi*, *I. redikorzevi, I. trianguliceps, Rhipicephalus fulvus*). However, the variety of species parasitizing rodents as immature stages is much higher [1].

The importance of hard-ticks in the epidemiology of several human vector-borne infections has received considerable attention in recent years and will certainly offer an opportunity for new studies in the years to come. The ecology of tick-borne infections is a popular field in parasitology and besides the research focused on the molecular epidemiology of tick-borne pathogens, studies on host preferences, seasonal variation and community structure are nevertheless important. From their reservoir-host perspective, rodents are known to act as key ecological links in the very complex transmission chains of tick-borne diseases as Lyme borreliosis or viral encephalitis [1,4].

Romania has an outstanding position in terms of biodiversity, being the only European country with five ecoregions on its territory [5]. This unique situation created a wide range of habitats and is mirrored by the number of mammal species present (112 species) [6]. Moreover, Romania not only holds this high biodiversity (especially among rodents [7]), but has nearly half of its human population living and working in rural areas and maintaining close contacts with nature [8], creating an interesting situation for epidemiological processes. Thirty-two species of wild rodents are known to occur in Romania [6]. Both this habitat variety and available host diversity [9] account for relatively high tick species diversity in Romania (25 species) [10], as compared to neighbouring countries [11]. However, micromammal-tick associations have been poorly studied in Romania despite the importance of each in the ecology of public pathogens. In this context, our manuscript shows the results of a study of tick infestation epidemiology in rodents from Romania.

## Methods

**Figure 1:**
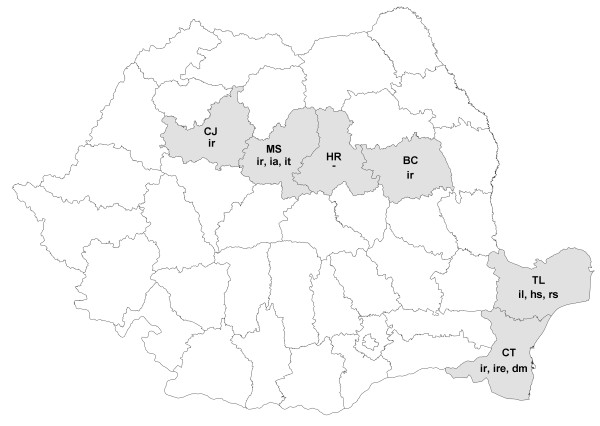
**Geographical distribution of ticks collected from rodents (county names: BC - Bacău, CJ - Cluj, CT - Constanţa, HR - Harghita, MS - Mureş, TL - Tulcea; tick species: dm - *Dermacentor marginatus***, **hs -*Haemaphysalis sulcata*, ia -*Ixodes apronophorus*, il -*Ixodes laguri*, ire -*Ixodes redikorzevi*, ir -*Ixodes ricinus*, it -*Ixodes trianguliceps*, rs -*Rhipicephalus sanguineus*).**

423 rodents from 12 species (Table 1) were collected from a variety of habitats in Romania between May 2010 and November 2011 (Figure 1). Rodents were caught using overnight snap-traps with peanut butter or chocolate bait. The traps were controlled early in the morning and the captured animals were immediately transferred to individual plastic zip bags and frozen. Each individual rodent was carefully checked for the presence of ectoparasites under a dissection microscope in the laboratory. All collected ticks were fixed in 70% ethanol for subsequent examination. Identification to species level was done according to morphological keys [12,13]. Identification of rodent species was carried out according to Aulaigner *et al*. 2009 [14]. Digital maps were created using ArcGis/ArcMap 9.2 (ESRI, © 1999–2006). The following epidemiological parameters were calculated: prevalence (per cent of infested animals from the total number of examined animals), mean intensity (total number of ticks collected per total number of infested animals) and mean abundance (total number of ticks collected per total number of examined animals) [15]. Frequency, prevalence and its 95% confidence intervals were calculated using the EpiInfo 2000 software. A *p* value of <0.05 was considered statistically significant. 

**Table 1:** Rodent species collected (total number, number by county and by month)

**Species**	**By County**	**By Month**
*Apodemus agrarius* (n=94)	Buzău (n=2) Cluj (n=72) Constanţa (n=3) Mureş (n=17)	April (n=5) May (n=4) August (n=3) September (n=27) October (n=47) December (n=8)
*Apodemus flavicollis* (n=51)	Bacău (n=1) Cluj (n=17) Mureş (n=28) Tulcea (n=5)	April (n=4) May (n=8) August (n=12) September (n=6) October (n=15)
*Apodemus sylvaticus* (n=22)	Cluj (n=8) Constanţa (n=10) Mureş (n=3) Tulcea (n=1)	April (n=3) May (n=3) June (n=1) September (n=2) October (n=10) December (n=3)
*Apodemus uralensis* (n=24)	Constanţa (n=18) Harghita (n=2) Mureş (n=2) Tulcea (n=2)	April (n=5) May (n=2) October (n=17)
*Myodes glareolus* (n=32)	Cluj (n=6) Mureş (n=26)	May (n=2) August (n=7) October (n=23)
*Micromys minutus* (n=11)	Cluj (n=7) Constanţa (n=3) Tulcea (n=1)	April (n=1) July (n=1) October (n=8) December (n=1)
*Microtus arvalis* (n=54)	Cluj (n=5) Constanţa (n=39) Mureş (n=10)	April (n=1) May (n=4) June (n=2) August (n=3) September (n=1) October (n=41) November (n=1) December (n=1)
*Microtus subterraneus* (n=49)	Cluj (n=44) Harghita (n=1) Mureş (n=4)	May (n=5) June (n=1) August (n=1) September (n=21) October (n=18) December (n=5)
*Mus musculus* (n=53)	Cluj (n=47) Harghita (n=5) Mureş (n=1)	Aprilie (n=3) May (n=2) June (n=1) August (n=2) September (n=25) October (n=15) November (n=5)
*Mus spicilegus* (n=8)	Bacău (n=1) Cluj (n=1) Constanţa (n=1) Tulcea (n=5)	April (n=2) July (n=5) September (n=1)
*Rattus norvegicus* (n=12)	Cluj (n=10) Harghita (n=1) Mureş (n=1)	April (n=1) June (n=1) July (n=1) September (n=1) October (n=5) November (n=3)
*Spermophilus citellus* (n=13)	Constanţa (n=1) Tulcea (n=12)	

## Results

From the total of 423 examined animals, 125 (29.55%) harboured ticks with a mean intensity of 3.86 and a mean abundance of 1.14 (Table 2). The highest prevalence of tick infestation was found in *Microtus arvalis* (70.37%) while two species did not harbour ticks at all (*Mus musculus*, *Rattus norvegicus*). The highest intensity was found in *Apodemus agrarius* (7.10) and the highest mean abundance in *M. arvalis* (2.87).

**Table 2:** Prevalence, intensity and abundance of hard-tick parasitism in rodents by host species

**Host**	**Examined (n)**	**With ticks (n)**	**Prevalence (%)**	**Intensity (range; mean±sd)**	**Abundance (mean±sd)**
*Apodemus agrarius*	94	21	22.34	1-67; 7.10±14.16	1.59±7.21
*Apodemus flavicollis*	51	26	50.98	1-12; 3.65±3.24	1.86±2.94
*Apodemus sylvaticus*	22	4	18.18	1-5; 2.50±1.91	0.45±1.22
*Apodemus uralensis*	24	13	54.17	1-6; 2.69±1.97	1.46±1.98
*Myodes glareolus*	32	16	50.00	1-4; 1.69±1.01	0.84±1.11
*Micromys minutus*	11	2	18.18	1; 1.00±0.00	0.18±0.40
*Microtus arvalis*	54	38	70.37	1-25; 4.08±4.25	2.87±4.01
*Microtus subterraneus*	49	2	4.08	2; 2.00±0.00	0.08±0.40
*Mus musculus*	53	0	0.00	-	-
*Mus spicilegus*	8	1	12.50	1; 1.00±0.00	0.13±0.35
*Rattus norvegicus*	12	0	0.00	-	-
*Spermophilus citellus*	13	2	15.38	1-4; 2.50±2.12	0.38±1.12
**Total**	**423**	**125**	**29.55**	**1-67; 3.86±6.58**	**1.14±3.98**

The total number of ticks collected from rodents was 483, with eight species identified (Table 3). The dominant species was *I. ricinus* (71.01%), followed by *I. redikorzevi* (23.60%) and *I. apronophorus* (2.48%). The other 5 species accounted each for less than 1.5% from the total of the collected ticks. The majority of *I. ricinus* collected were larvae (76.97%), while in case of *I. redikorzevi,* nymphs were predominant (82.46%).

**Table 3:** Developmental stage distribution of ticks feeding on rodents in Romania (number and percentage of all collected)

**Tick species**	**Total number of ticks**	**Adults**	**Nymphs**	**Larvae**
*Ixodes ricinus*	343 (71.01)	16 (4.66)	63 (18.37)	264 (76.97)
*Ixodes redikorzevi*	114 (23.60)	20 (17.54)	94 (82.46)	0 (0.00)
*Ixodes laguri*	1 (0.21)	1 (100)	0 (0.00)	0 (0.00)
*Ixodes apronophorus*	12 (2.48)	0 (0.00)	0 (0.00)	12 (100)
*Ixodes trianguliceps*	2 (0.41)	1 (50.00)	0 (0.00)	1 (50.00)
*Dermacentor marginatus*	1 (0.21)	1 (100)	0 (0.00)	0 (0.00)
*Rhipicephalus sanguineus*	6 (1.24)	0 (0.00)	2 (33.33)	4 (66.67)
*Haemaphysalis sulcata*	4 (0.83)	0 (0.00)	0 (0.00)	4 (100)
**Total**	483 (100)	39 (8.07)	159 (32.92)	285 (59.01)

The highest overall prevalence was recorded for *I. ricinus* (20.57% of rodents infested) followed by *I. redikorzevi* (7.09%). All other ticks species had prevalences below 0.5% (Table 4). Only two hosts had polyspecific parasitism, with *I. ricinus* + *I. redikorzevi* and *I. ricinus* + *Dermacentor marginatus* respectively.

**Table 4:** Prevalence of developmental stages by tick species (number and percentage of all collected)

**Tick species**	**Number of rodents infested**	**Host with adults**	**Host with nymphs**	**Host with larvae**
*Ixodes ricinus*	87 (20.57)	6 (6.90)	28 (32.18)	64 (73.56)
*Ixodes redikorzevi*	30 (7.09)	12 (40.00)	23 (76.67)	0 (0.00)
*Ixodes laguri*	1 (0.24)	1 (100.0)	0 (0.00)	0 (0.00)
*Ixodes apronophorus*	2 (0.47)	0 (0.00)	0 (0.00)	2 (100)
*Ixodes trianguliceps*	1 (0.24)	1 (100)	0 (0.00)	1 (100)
*Dermacentor marginatus*	1 (0.24)	1 (100)	0 (0.00)	0 (0.00)
*Rhipicephalus sanguineus*	2 (0.47)	0 (0.00)	2 (100)	1 (50.00)
*Haemaphysalis sulcata*	1 (0.24)	0 (0.00)	0 (0.00)	1 (100)
**Total**	125 (29.55)*	**21 (16.80)**	**53 (42.40)**	**69 (55.20)**

*2 animals with polyspecific infestation.

The highest number of host species was recorded for *I. ricinus* (8 host species) followed by *I. redikorzevi* (3 host species) and *Rhipicephalus sanguineus* (2 host species). All the other tick species were found only on a single host species (Table 5). Adult ticks (regardless of the species) were found on 5 host species, nymphs on 6 host species and larvae on 7 species (Table 5).

**Table 5:** Tick-rodent associations in Romania

**Tick species**	**Hosts for adults**	**Hosts for nymphs**	**Hosts for larvae**	**Host species**
*Ixodes ricinus*	*Aa, Mm, Ma*	*Aa, Af, As, Au, Ma*	*Aa, Af, As, Au, Mg, Ma, Msu*	*Aa, Af, As, Au, Ma, Mg, Mm, Msu*
*Ixodes redikorzevi*	*Au, Ma, Mm*	*Au, Ma*	-	*Au, Ma, Mm*
*Ixodes laguri*	*Sc*	-	-	*Sc*
*Ixodes apronophorus*	-	-	*Af*	*Af*
*Ixodes trianguliceps*	*Msu*	-	*Msu*	*Msu*
*Dermacentor marginatus*	*Ma*	-	-	*Ma*
*Rhipicephalus sanguineus*	-	*Af, Msp*	*Af*	*Af, Msp*
*Haemaphysalis sulcata*	-	-	*Sc*	*Sc*
**Total**	*Aa, Mm, Ma, Msu, Sc*	*Aa, Af, As, Au, Ma, Msp*	*Aa, Af, As, Au, Mg, Ma, Msu*	

Aa - Apodemus agrarius; Af - Apodemus flavicollis; As - Apodemus sylvaticus; Au - Apodemus uralensis; Mg - Myodes glareolus; Mm - Micromys minutus; Ma - Microtus arvalis; Msu - Microtus subterraneus; Msp - Mus spicilegus; Sc - Spermophilus citellus.

The regional distribution of ticks parasitizing rodents shows that certain species were found in both examined regions (i.e. *I. ricinus* central and south-eastern Romania), while others were restricted to the central part (*I. apronophorus*, *I. trianguliceps*) or the south-eastern part (*I. laguri*, *Haemaphysalis sulcata*, *R. sanguineus*, *I. redikorzevi*) (Figure 1).

## Discussion

### Host preferences

In the case of Lyme borreliosis, small mammals are the vertebrate group that has been the most extensively investigated up to now, mainly because they can be easily captured in large numbers, handled and maintained in the laboratory [2]. The main reservoir hosts for *Borrelia burgdorferi sensu lato* (s.l.) in Europe are *A. agrarius*, *A. flavicollis*, *A. sylvaticus* and *Myodes glareolus*. Moreover, certain genospecies of this pathogen (i.e. *Borrelia afzelii*) are cycled almost exclusively by rodents [2]. The ecological importance of reservoir hosts is greater if they are also common hosts to competent vector ticks. For instance, several vertebrate species were experimentally demonstrated to be competent reservoir hosts but their role as hosts to competent vector ticks is less important (i.e. *R. norvegicus*, *R. rattus*, *Sciurus vulgaris*, *Glis glis*[2]. Our study suggests that certain rodent species are more prone to be attacked by ticks than others. In species like *M. arvalis*, *A. uralensis*, *A. flavicollis* and *M. glareolus* the overall prevalence of parasitism with hard ticks was more than 50%. On the other hand, we found lower prevalence in *A. agrarius*, *A. sylvaticus*, *Micromys minutus*, *Mus spicilegus* and *Spermophilus citellus* even if sympatric with other infested hosts species. Interestingly, very abundant synanthropic rodent species like *M. musculus* and *R. norvegicus* were not harbouring ticks at all.

In a similar study from France, the overall prevalence of tick burden in micromammals was 25.19%, with *I. ricinus* being the dominant tick-parasite [16]. The authors found the highest prevalence in *M. arvalis* (31.58%), followed by *A. sylvaticus* (22.73%), *M. agrestis* (16.13%) and *M. glareolus* (14.16%). In the Netherlands [17], variable prevalences (19-56%) of tick parasitism in *A. sylvaticus* were reported during spring and summer and the only tick species found was *I. ricinus*. It seems also that the most important reservoir hosts for the Lyme borreliosis agent are usually infested with a higher number of ticks than other rodent species. Higher mean intensity and abundance were found in *A. agrarius*, *A. flavicollis*, *A. sylvaticus*, *A. uralensis* and *M. arvalis* while in other host species these parameters were lower (i.e. *Mus spicilegus*, *Micromys minutus*).

### Community and population structure

Another important aspect is the tick species diversity found in our study. Most published data on ticks of rodents from Europe report few species. A survey on 799 micromammals in France revealed the presence of only two tick species: *I. ricinus* and *I. trianguliceps*[16]. In the Netherlands, only *I. ricinus* was reported from rodents [16], while in rodents from Russia four tick species were found [18]. In a multinational study (Germany, Slovakia and Romania) on the epidemiology of TBE virus, the authors reported only *I. ricinus* on *A. flavicollis*, *A. sylvaticus*, *A. uralensis* and *M. glareolus* and *I. trianguliceps* on *Microtus subterraneus*[19]. In a study from Germany, out of 11,680 ticks collected from rodents (*A. flavicollis*, *A. sylvaticus* and *M. glareolus*), 97.9% were *I. ricinus, while* the rest were *I. trianguliceps*[20].

All these data, together with other nation-wide surveys [21] add new evidence that the principal tick infesting rodents in Europe is mainly *I. ricinus*. *Ixodes ricinus* is also the most common tick feeding on humans [22], which may confer to rodents an important status as reservoir hosts for human diseases [23].

The host sharing by different tick species is important mainly for the bridging of microbial pathogens through the reservoir hosts. Although ticks specifically feeding on rodents (i.e. *I. apronophorus*, *I. redikorzevi*, *I. trianguliceps*) are attacking humans only exceptionally [24], they may maintain the infection cycle of their rodent host with certain pathogens. Subsequently, a more generalist tick (usually *I. ricinus*) can bridge the pathogens from these rodents to humans. Examples include *B. burgdorferi* s.l. isolated from *I. trianguliceps*[25] and *I. redikorzevi*[26] or the Omsk virus isolated from *I. apronophorus*[27], all in Russia.

Assessing the age structure of tick populations infesting rodents, using the prevalence of each developmental stage showed a skewed age ratio towards immatures. In Germany, a study of the population structure of *I. ricinus* on three rodent species showed that 97.9% of all ticks were larvae, 2.0% nymphs, and 0.1% females [20]. A multinational study focusing on rodents' ticks in Central Europe found only larvae and nymphs [19]. In the case of *I. ricinus*, our study confirmed other general observations [13], according to which rodents are important hosts mainly for the immature stages of this tick. Although in our study we found adults of *I. ricinus* on 1.4% of the examined animals, interestingly, the majority of them were collected from *M. arvalis*. From 54 examined animals, four (7.4%) harboured adults of *I. ricinus*. This suggests that certain rodent species can act also as more common hosts for *I. ricinus*.

### Geographical distribution

According to a recent review [10], a number of tick species found in the present study have a widespread distribution in Romania (*I. ricinus*, *D. marginatus*), while others are restricted to the southern regions (*I. laguri*, *H. sulcata*, *R. sanguineus*). The results of tick community structures from rodents analysed in accordance with general distribution maps [10] show that rodents are a good marker for assessing the distribution of certain tick species, but more heterogeneous seasonal collection campaigns are required to draw reliable conclusions.

## Conclusions

Our study showed a relatively high diversity of ticks parasitizing rodents in Romania. The most common tick in rodents was *I. ricinus*, followed by *I. redikorzevi*. Certain rodents seem to host a significantly higher number of tick species than others, the most important within this view being *Apodemus flavicollis* and *Microtus arvalis*. The same applies for the overall prevalence of tick parasitism, with some species more commonly infected (*M. arvalis*, *A. uralensis*, *A. flavicollis* and *M. glareolus*) than others. Two rodent species (*Mus musculus*, *Rattus norvegicus*) did not harbour ticks at all. Based on our results we may assert that rodents generally can act as good indicators for assessing the distribution of certain tick species.

## Competing interests

All authors have seen and approved the manuscript and declare that they have no competing interest.

## Authors’ contributions

MAD conceived the study and drafted the manuscript. DMA and MC identified the ticks. SDA contributed to study design and identified the small mammals. OM, MIA and IA examined the rodents and collected the ticks. GA performed the data analysis. DG collected the samples in the field. CV is the team coordinator, while GCM designed the study and coordinated the research grant. All authors read and approved the final manuscript.

## Financial support

This study was supported by a research grant from the CNCSIS (84, 7/2010).

## References

[B1] Durden LA Taxonomy, host associations, life cycles and vectorial importance of ticks parasitizing small mammals 2006 Micromammals and Macroparasites From Evolutionary Ecology to Management, Springer-Verlag Tokyo: In S. Morand, B.R. Krasnov, R. Poulin (Eds.) 91 102

[B2] Gern L Humair PF Ecology of Borrelia burgdorferi sensu lato in Europe 2002 CABI: In Lyme Borreliosis: Biology, Epidemiology and Control (Gray JS, Kahl O, Lane RS, Stanek G, editors) 149 174

[B3] Dantas-Torres F Latrofa MS Otranto D Quantification of Leishmania infantum DNA in females, eggs and larvae of Rhipicephalus sanguineus Parasit Vectors 2011 4 56 10.1186/1756-3305-4-56 21489252 PMC3094396

[B4] Kurtenbach K Schäfer SM de Michelis S Etti S Sewell HS Borrelia burgdorferi sensu lato in the vertebrate host 2002 ICABI: n Lyme Borreliosis: Biology, Epidemiology and Control (Gray JS, Kahl O, Lane RS, Stanek G, eds) 117 148

[B5] Cogălniceanu D Cogălniceanu CG An enlarged European Union challenges priority settings in conservation Biodiv Conserv 2010 19 1471 1483 10.1007/s10531-010-9777-1

[B6] Popescu A Murariu D [Fauna of Romania: Mammalia Vol. XVI., Fasc. 2: Rodentia] 2001 [in Romanian]: Editura Academiei

[B7] Krystufek B Griffiths HI Species richness and rarity in European rodents Ecography 2002 25 120 128 10.1034/j.1600-0587.2002.250114.x

[B8] Vincze M Kerekes K Impact of CAP’s Pillars on Romanian Rural Employment 2009 Debrecen: Proceedings of the Aspects and Visions of Applied Economics and Informatics Conference 1338 1351

[B9] Doniţă N Popescu A Paucă-Comănescu M Mihăilescu S Biriş IA Habitats from Romania 2005 Bucureşti: Editura Tehnică Silvică [in Romanian]

[B10] Mihalca AD Dumitrache MO Magdaş C Gherman CM Domşa C Mircean V Ghira IV Pocora V Ionescu DT Sikó Barabási S, Cozma V, Sándor AD: Synopsis of the hard-ticks (Acari: Ixodidae) of Romania with update on host associations and geographical distribution Exp Appl Acarol 2012 58 183 206 10.1007/s10493-012-9566-5 22544174

[B11] Kolonin GV Fauna of ixodid ticks of the world (Acari, Ixodidae) 2009 http://www.kolonin.org

[B12] Feider Z [Fauna of the Popular Republic of Romania. Volume 5/2. Acaromorpha, Suprafamily Ixodoidea] 1965 [in Romanian]: Editura Academiei Republicii Populare Române, Bucuresti

[B13] Nosek J Sixl W Central-European ticks (Ixodoidea) Mitt Abt Zool Landesmus Joanneum 1972 1 61 92

[B14] Aulagnier S Haffner P Mitchell-Jones AJ Moutou F Zima J Mammals of Europe 2009 London: North Africa and the Middle East. A&C Black

[B15] Rózsa L Reiczigel J Majoros G Quantifying parasites in samples of hosts J Parasitol 2000 86 228 232 10780537 10.1645/0022-3395(2000)086[0228:QPISOH]2.0.CO;2

[B16] L'Hostis M Dumon H Fusade A Lazareff S Gorenflot A Seasonal incidence of Ixodes ricinus ticks (Acari: Ixodidae) on rodents in western France Exp Appl Acarol 1996 20 359 356 10.1007/BF00130548 8771769

[B17] de Boer R Hovius KE Nohlmans MK Gray JS The woodmouse (Apodemus sylvaticus) as a reservoir of tick-transmitted spirochetes (Borrelia burgdorferi) in the Netherlands Zentralbl Bakteriol 1993 279 404 416 10.1016/S0934-8840(11)80373-7 8219511

[B18] Panina TV Katelina AF Ixodid ticks as parasites of the common red-backed vole (Clethrionomys glareolus) 1993 Tula: In Problems of Natural Focal Infections and Medical Geography Conference (Demianov AG et al. eds.) 69 72

[B19] de Mendonça PG Benedek AM Jurčovičová M Molecular screening of European wild rodents for tick-borne encephalitis virus Acta Zool Bulgar 2011 63 2 195 197

[B20] Kurtenbach K Kampen H Dizij A Arndt S Seitz HM Schaible UE Simon MM Infestations of rodents with larval Ixodes ricinus (Acari, Ixodidae) is an important factor in the transmission cycle of Borrelia burgdorferi s.l. in German woodlands J Med Entomol 1995 32 807 817 8551503 10.1093/jmedent/32.6.807

[B21] Mihalca AD Gherman CM Magdaş C Dumitrache MO Györke A Sándor AD Domşa C Oltean M Mircean V Mărcuţan DI D’Amico G Păduraru AO Cozma V Ixodes ricinus is the dominant questing tick in forest habitats from Romania: the results from a countrywide dragging campaign Exp Appl Acarol 2012 58 2 175 182 10.1007/s10493-012-9568-3 22547023

[B22] Briciu VT Titilincu A Ţăţulescu DF Cârstina D Lefkaditis M Mihalca AD First survey on hard ticks (Ixodidae) collected from humans in Romania: possible risks for tick-borne diseases Exp Appl Acarol 2011 54 2 199 204 10.1007/s10493-010-9418-0 21161719

[B23] Schorn S Pfister K Reulen H Mahling M Silaghi C Occurrence of Babesia spp., Rickettsia spp. and Bartonella spp. in Ixodes ricinus in Bavarian public parks, Germany Parasit Vectors 2011 4 135 10.1186/1756-3305-4-135 21762494 PMC3154157

[B24] Bursali A Tekin S Orhan M Keskin A Ozkan M Ixodid ticks (Acari: Ixodidae) infesting humans in Tokat Province of Turkey: species diversity and seasonal activity J Vector Ecol 2010 35 180 186 10.1111/j.1948-7134.2010.00075.x 20618665

[B25] Gorelova NB Korenberg EI Kovalevskii YV Postic D Baranton G Isolation of Borrelia from the tick Ixodes trianguliceps (Ixodidae) and the significance of this species in epizootiology of ixodid tick-borne borreliosis Parazitologiya 1996 30 13 18 [in Russian] 8975209

[B26] Rigó K Miklós G Tóth AG Földvari G Detection of Borrelia burgdorferi sensu lato and Anaplasma phagocytophilum in small mammals and ectoparasites in Hungary Vector-Borne Zoonot 2011 11 1499 1501 10.1089/vbz.2011.0608 21736488

[B27] Eldridge BF Scott TW Day JF Tabachnick WJ Arbovirus Diseases 2004 Kluwer Academic Publishers: In Medical Entomology - A Textbook on Public Health and Veterinary Problems Caused by Arthropods, (Eldridge BF, Edman JD Eds.) 415 460

